# Genome-Wide Analysis of the Xyloglucan Endotransglucosylase/Hydrolase (*XTH*) Gene Family: Expression Pattern during Magnesium Stress Treatment in the Mulberry Plant (*Morus alba* L.) Leaves

**DOI:** 10.3390/plants13060902

**Published:** 2024-03-21

**Authors:** Blessing Danso, Michael Ackah, Xin Jin, Derek M. Ayittey, Frank Kwarteng Amoako, Weiguo Zhao

**Affiliations:** 1 Jiangsu Key Laboratory of Sericulture Biology and Biotechnology, School of Biotechnology, Jiangsu University of Science and Technology, Zhenjiang 212100, China; blessingdanso@hotmail.com (B.D.);; 2 School of the Environment and Safety Engineering, Jiangsu University, Zhenjiang 212013, China; 3 School of Food and Biological Engineering, Jiangsu University, Zhenjiang 212013, China; 4 Key Laboratory of Silkworm and Mulberry Genetic Improvement, Ministry of Agriculture and Rural Affairs, The Sericultural Research Institute, Chinese Academy of Agricultural Sciences, Zhenjiang 212100, China; 5 School of Fisheries and Life Sciences, Shanghai Ocean University, Shanghai 201308, China; 6 Institute of Plant Nutrition and Soil Science, Kiel University, Hermann-Rodewald-Straße 2, 24118 Kiel, Germany; kwamekwarteng242@gmail.com

**Keywords:** *Morus alba*, xyloglucan endotransglucosylase/hydrolase (*XTH*), phylogeny, magnesium stress, expression patterns

## Abstract

Mulberry (*Morus alba* L.), a significant fruit tree crop, requires magnesium (Mg) for its optimal growth and productivity. Nonetheless, our understanding of the molecular basis underlying magnesium stress tolerance in mulberry plants remains unexplored. In our previous study, we identified several differential candidate genes associated with Mg homeostasis via transcriptome analysis, including the xyloglucan endotransglucosylase/hydrolase (*XTH*) gene family. The *XTH* gene family is crucial for plant cell wall reconstruction and stress responses. These genes have been identified and thoroughly investigated in various plant species. However, there is no research pertaining to *XTH* genes within the *M. alba* plant. This research systematically examined the *M*. *alba XTH* (*MaXTH*) gene family at the genomic level using a bioinformatic approach. In total, 22 *MaXTH* genes were discovered and contained the Glyco_hydro_16 and XET_C conserved domains. The *MaXTH*s were categorized into five distinct groups by their phylogenetic relationships. The gene structure possesses four exons and three introns. Furthermore, the *MaXTH* gene promoter analysis reveals a plethora of cis-regulatory elements, mainly stress responsiveness, phytohormone responsiveness, and growth and development. GO analysis indicated that *MaXTH*s encode proteins that exhibit xyloglucan xyloglucosyl transferase and hydrolase activities in addition to cell wall biogenesis as well as xyloglucan and carbohydrate metabolic processes. Moreover, a synteny analysis unveiled an evolutionary relationship between the *XTH* genes in *M*. *alba* and those in three other species: *A*. *thaliana*, *P*. *trichocarpa*, and *Zea mays*. Expression profiles from RNA-Seq data displayed distinct expression patterns of *XTH* genes in *M*. *alba* leaf tissue during Mg treatments. Real-time quantitative PCR analysis confirmed the expression of the *MaXTH* genes in Mg stress response. Overall, this research enhances our understanding of the characteristics of *MaXTH* gene family members and lays the foundation for future functional genomic study in *M*. *alba.*

## 1. Introduction 

Mulberry (*Morus* spp.) is an essential plant in many Chinese provinces, primarily cultivated for its leaves and fruits. It holds particular significance in sericulture, as mulberry leaves serve as the exclusive food source for domestic silkworms (*Bombyx mori* L.) [1,2]. Besides its historical role in silkworm rearing, mulberry, especially *Morus alba* (*M*. *alba*), demonstrates potential as a pioneer tree species in marginal environments [3]. Moreover, the leaves of *M*. *alba* are of high medicinal value [4] and are believed to possess antioxidant, anti-inflammatory, and anti-allergic properties attributed to various bioactive phytochemicals, including polyphenolic compounds, triterpenoids, and anthocyanins. Although *M*. *alba* is of significant economic importance, its growth and development are subject to the influence of nutrient concentrations. High magnesium (Mg) levels or Mg deficiency are among the key factors affecting the growth and development of the plant [5]. Nevertheless, *M*. *alba*’s stress response to different Mg concentrations is unclear, especially at the genomic level.

Mg deficiency frequently hinders crop yield in sandy or highly acidic soils, primarily attributable to the high leaching susceptibility of Mg. This occurrence is widely observed and has notable implications for agricultural productivity in such soil conditions [6]. Extensive investigations have scrutinized and unveiled the consequences of Mg deficiency on plant physiological aspects, including biomass distribution, carbon dioxide (CO_2_) uptake, and protection against photooxidative stress [7], resulting in yield reduction and poor fruit quality [8,9]. In response to these challenges, plants have developed intricate regulatory mechanisms, including the involvement of distinct gene families, such as Xyloglucan endotransglucosylase/hydrolases (*XTH*s) [10]. *XTH*s genes are classified within the glycoside hydrolase family 16 and are an essential group of enzymes primarily responsible for cleaving and rearranging the xyloglucan backbones within plant cell walls [11,12,13]. Specifically, family members of this gene carry out two distinct biochemical processes that are catalyzed by two specific enzymes: xyloglucan endotransglycosylase (*XET*) and xyloglucan endohydrolase (*XEH*) [14]. XET catalyzes the transfer of one xyloglucan molecule to another, resulting in the elongation of xyloglucan, whereas *XEH* is characterized by hydrolyzing an individual xyloglucan molecule, causing an irreversible reduction in the length of the xyloglucan chain [13].

Several *XTH*s exhibit catalytic properties and play an essential role in regulating the extensibility of plant cell walls, root elongation, and plant growth [11,15]. Due to the advancement of sequencing technology and data availability, an expanding repertoire of *XTH* genes has been discovered and characterized in a broader range of species including *Ananas comosus* (48) [16], *Arachis hypogaea* L. (58) [17], *Glycine max* (61) [18] *Arabidopsis thaliana* [19], *lpomoea batatas* (36) [20], *Oryza sativa* (29) [21], *Solanum lycopersicum* L. (37) [11], *Nicotiana tabacum* (56) [22] *Brassica rapa* (53) and *Brassica oleracea* (35) [23]. Nonetheless, the *XTH* family constituents in mulberry remain undisclosed. Earlier investigations have demonstrated the involvement of *XTH* genes in numerous crucial processes, particularly the development and growth of plants via the remodelling of plant cell walls. For instance, in *Arabidopsis*, genes such as *AtXTH17*, *AtXTH18*, *AtXTH19*, and *AtXTH20* exhibited specific expression patterns in the root tissues and were significantly involved in the processes of root elongation and the initiation of root hair formation [19,24]. *GhXTH1* gene overexpressed in cotton, elongated cotton fibers by 15–20% [25]. In addition, some *XTH* genes have been reported to play active roles in fruit softening and ripening. *PavXTH14* and *PavXTH15* expression in cherry fruits resulted in a significant decrease in fruit firmness and altered the constitution of hemicellulose and pectin in the cell wall of the transgenic fruit [26]. Likewise, *XTH* influenced the softening and ripening of fruits, including tomatoes [27], strawberries [28], kiwi [29], and pears [29]. Several other *XTH* genes are involved in flower development [29] and leaves [30].

Numerous investigations have also suggested that plant hormones play a role in regulating the activity of *XTH* genes. For instance, the application of abscisic acid increased the expression of *Arabidopsis AtXTH23* [19]. Similarly, the ethylene application induced the expression of banana *MA-XETI*, which is involved in the ripening and softening of the peel and pulp [30]. Furthermore, under ethylene induction, three *CaXTH* genes were significantly upregulated in the leaf tissue of hot pepper [31]. Members of the *XTH* gene family primarily regulate cell wall responses to biotic and abiotic stressors, which affect plant growth. The overexpression of *DkXTHI* was found to augment the resistance of transgenic *Arabidopsis* plants to salt, drought-induced stress, and abscisic acid, consequently impacting the development of roots and leaves [32]. Similarly, *XTH* genes in Chinese cabbage (*Brassica rapa* L.) exhibited an upregulated expression in response to elevated temperatures [33]. Furthermore, under low temperatures, the *DkXTH6* gene in persimmons decreased in expression, while the *DkXTH7* gene showed noticeably high transcription levels [34]. A prior proteomic study in maize revealed that *XTH*s were differently regulated in response to drought stress [35]. Moreover, xyloglucan content was decreased in the *Arabidopsis AtXTH31* mutant, which lowered the amount of absorbed Al^3+^ and increased resistance to aluminum stress [36]. The overexpression of the xyloglucan endotransglucosylase/hydrolase gene in *Populus euphratica* resulted in increased resistance to cadmium tolerance by limiting cadmium absorption in the root system of transgenic tobacco plants. In addition, the transgenic plants had 56–87% more xyloglucan degrading activity (XDA) than the wild type, which resulted in a 25–27% decrease in the amount of xyloglucan in the root cell walls [37]. Moreover, in *Arabidopsis*, aluminum tolerance was imparted by the induction of *ZmXTH*, a gene encoding xyloglucan endotransglucosylase/hydrolase from maize [38]. Similarly, *Arabidopsis* mutants *xth15* and *xthI7* exhibited elevated aluminum tolerance in contrast to wild-type plants [36].

These preliminary studies highlight the key role of *XTH*s in various plants’ responses to various stresses. However, to the best of our knowledge, there is no functional characterization of mulberry *XTH* gene family members. Consequently, there is a necessity for a systemic and comprehensive exploration of the *M*. *alba XTH* gene family across the genome. The present investigation conducted an analysis of the *XTH* gene family within *M*. *alba* based on our previous transcriptomic analysis after Mg stress treatment using the available genome data. Subsequently, detailed information, including phylogenetic analysis, gene structure characterization, chromosomal localization, motif analysis, promoter analysis, and syntenic relationships of *MaXTH* genes, were examined. Furthermore, real-time quantitative PCR (qRT-PCR) was employed to determine the expression patterns of *XTH* genes in the leaf tissues of *M*. *alba* that were subjected to various levels of Mg stress. The findings of this study are poised to offer significant insights into the *XTH* genes in *M*. *alba*, contributing to deeper comprehension and setting the groundwork for the functional analysis of plant *XTH* genes in mulberry plants.

## 2. Materials and Methods

### 2.1. Growth Conditions and Magnesium Treatment for the Mulberry Plant (Morus alba)

Mulberry (Yu-711), a member of the *M*. *alba* species, was obtained from the National Mulberry GenBank at Jiangsu University of Science and Technology in Zhenjiang, Jiangsu, China. Growth of the mulberry plant materials and Mg treatments followed the methods of our previous study [5]. In brief, mulberry seedlings that had been grafted were carefully chosen and then planted in pots with a diameter of 35 cm. These pots included a mixture of loamy soil and vermiculite. The optimal circumstances for the plant’s growth in the greenhouse consist of 14 h of light, 10 h of darkness, a temperature of 25 °C during the day and 20 °C at night, and a humidity level ranging from 70 to 80%. A total of 18 pots were utilized, with each pot housing three plant seedlings as duplicates. The seedlings were watered daily and provided with a solution of MS culture medium consisting of 4.37 g of MS media dissolved in 1000 mL of water (pH = 7.0) every three days for a total of 7 days. The plants were subjected to a 7-day treatment with deionized water following complete leaf growth. Mg (MgSO_4_) treatments were then administered. A total of six concentration gradients, 0 mM (T1) as Mg deficiency, 1 mM and 2 mM (T2 and T3) as low Mg, 3 mM as sufficiency (CK), and 6 mM and 9 mM (T4 and T5) as Mg excess, were applied to the mulberry plants for 20 days. On the 20th day, leaves from all experimental groups and control were collected. Leaf samples harvested from the control and treated plants were wrapped separately in plain plastic bags and temporarily preserved (one week) at a temperature of −80 °C for subsequent studies, including transcriptome analysis [39].

### 2.2. Data Collection and Identification of XTH Gene Family Members in Morus alba

Genetic data in the form of genome sequences (fasta) and annotation files (gff) for three plant species, *Arabidopsis thaliana*, *Populus trichocarpa*, and *Zea mays*, were obtained from the official NCBI website (https://www.ncbi.nlm.nih.gov (accessed on 5 July 2023). *M*. *alba* genome sequence annotation file (fasta) was downloaded from NCBI (https://www.ncbi.nlm.nih.gov/datasets/genome/GCA_012066045.3/ (accessed on 7 July 2023)). However, the gff was obtained from Professor Weiguo Zhao of Jiangsu University of Science and Technology. To determine the *XTH* gene in the mulberry plant genome, an HMMER search was executed utilizing a hidden Markov model (HMM) profile comprising binding domains PF00722 and PF06955, sourced from the Pfam database (http://Pfam.xfam.org/ (accessed on 12 July 2023)). Sequences with E values < 1 were scrutinized, and any short open reading frames (less than 100 in length) were manually sorted out. The filtered sequences with putative of both PF00722 and PF06955 domains or either one of them were screened as primary candidates for *M. alba XTH* genes (*MaXTH*).

### 2.3. Gene Structure, Motif Analysis and Sequence Alignment

Each *MaXTH* gene structure was visualized in TBtools software v1.098769 [40] by utilizing the genome sequence in conjunction with its corresponding annotation file. Employing the online MEME suite (available at https://meme-suite.org/meme/ (accessed on 15 July 2023), the conserved motifs within *MaXTHs* were identified using specific parameters: a search for 10 motifs, with a minimum width of 6 and a maximum width of 55. The consensus motif sequence was conducted, and a web logo was generated through the utilization of the MEME tool. Subsequently, the individual motifs were searched in motif scan (https://myhits.sib.swiss/cgi-bin/motif_scan (accessed on 15 January 2024) to identify the Glyco_hydro_16 and XET_C domains. Furthermore, the extracted motif sequences of the *MaXTH* protein sequences exhibiting the Glyco_hydro_16 and XET_C domains were aligned in Bioedit software (v7.2).

### 2.4. Physicochemical Properties of the MaXTH Gene Family

The ExPASy online platform (https://web.expasy.org/protparam/ (accessed on 15 July 2023) was used to obtain the molecular weight (Mw), isoelectric point (pI), and grand average of hydropathy (GRAVY) data for individual XTH proteins. Additionally, the subcellular localization of MaXTH proteins was predicted using the CELLO online resource (http://cello.life.nctu.edu.tw/ (accessed on 25 July 2023) [41].

### 2.5. Phylogenetic Analysis

In the present investigation, two phylogenetic trees were constructed to categorize the *MaXTH* gene family. The first tree encompassed only the MaXTH protein sequences from *M*. *alba*, and the second tree included *M*. *alba*, *P. trichocarpa*, and *A. thaliana XTH* genes. The evolutionary relationships of the *XTH* genes in the different groups were assessed following the alignment of XTH protein sequences in MEGA 7 software using Clustal W [42]. A phylogenetic tree, based on the maximum likelihood method, with 1000 bootstrap replications was generated employing the MEGA 7.0 software (v7.0.26). All the phylogeny trees were performed following the same specifications indicated above.

### 2.6. Analysis of Cis-Regulatory Elements of MaXTH Genes and GO Analysis

The promoter regions located 2000 base pairs prior to the initiation codon (ATG) of *MaXTH* genes were extracted from the genome of *M*. *alba*. Utilizing the PlantCARE online tool (https://bioinformatics.psb.ugent.be/webtools/plantcare/html/ (accessed on 12 July 2023) [43], the inherent regulatory elements in these promoter regions were predicted. Subsequently, the outcomes of this predictive analysis were rendered visible through the TBtool software. Gene Ontology (GO) analysis was performed using the web tool Shiny GO (http://bioinformatics.sdstate.edu/go74/ (accessed on 2 August 2023).

### 2.7. Chromosomal Localization, Circos, and Synteny Analyses

The length of each chromosome and the chromosomal position of all *XTH* genes were obtained from the annotated dataset of the *M*. *alba* genome. The positional mapping of genes on the chromosomes was visualized through the utilization of TBtools software v1.098769 [40]. Based on the alignment and further examination of the phylogenetic relationship of the *MaXTH* genes, paralogous genes were discovered [20], and the interconnections of the paralogous genes according to their locations on the chromosomes were exhibited on the circos map constructed in the Tbtools (v2.026). The Multiple Collinearity Scan toolkit (MCScanX), integrated into the TBtool (v2.026), was applied for the identification of syntenic blocks and specific gene pairs, adhering to its default configurations [44]. Additionally, the Multiple Synteny Plotter program within the TBtool (v2.026) was employed to visually represent the synteny associations among orthologous *XTH* genes across *M*. *alba*, *A*. *thaliana*, *Zea mays*, and *Populus trichocarp* [45].

### 2.8. Gene Expression Analysis and qRT-PCR Gene Validation

By using the RNA-seq data obtained from our transcriptome analysis after Mg application (https://ncbi.nlm.nih.gov; accession number: PRJNA951543), this study examined the expression of the *XTH* genes in *M*. *alba* treated with different levels of Mg after 20 days (about 3 weeks) and compared it to the expression of the gene in optimum Mg supply (the control) according to our previous study [5]. To validate the *XTH* genes identified through high-throughput sequencing, six differentially expressed genes (DEGs) were chosen for real-time quantitative polymerase chain reaction (qRT-PCR) validation, following the established protocol detailed in a prior study [46]. Mulberry leaf samples used for the RNA-seq analysis were also used for total RNA and cDNA synthesis for qRT-PCR validation. Furthermore, the 2^−ΔΔCt^ method [47] was applied to estimate the fold changes in gene expression. The primer sequences and gene names for qRT-PCR validation are enlisted in Supplementary File S1.

## 3. Results

### 3.1. Identification and Physiological Features XTH Genes in M. alba

In our previous investigation, RNA-Seq transcriptome analysis of *M*. *alba* leaf tissues was conducted to ascertain candidate genes possibly influenced by high magnesium (Mg) levels or magnesium nutrient starvation. A comprehensive examination of the transcriptome data resulted in the identification of 12 *XTH* genes. Among them, the candidate *MaXTH-1* with the gene ID LOC21405692 had a higher expression level and, therefore, was selected and used as a query gene for the search against the *Morus* genome using BLASTP and HMMER search. Twenty-four putative *XTH* proteins were obtained after the search and confirmed in the *M. alba* genome using their transcript ID. After which, the short and redundant sequences underwent manual scrutiny. Subsequently, the identification of conserved domains was carried out utilizing the Pfam and CDD databases. Finally, the 22 *XTH* proteins obtained were named *MaXTH-1* to *MaXTH-22* (Table 1 and Supplementary File S2). Coding sequences (CDS) of the 22 *MaXTH* proteins ranged from 645 bp (LOC21407360; *MaXTH-21*) to 1509 bp (LOC21403517; *MaXTH-18*), with an average length of 915 bp. The amino acid sequence length of the *MaXTH* proteins spanned from 214–502, where *MaXTH-18* was the longest sequence (501 aa), and *MaXTH-21* (214 aa) had the shortest sequence. The molecular weights of MaXTH proteins varied between 24.071 kDa (*MaXTH-21*) and 56.843 kDa (*MaXTH-18*), averaging 34.40 kDa. The GRAVYs for all *MaXTH* genes were negative, ranging from −1003 to −0.099 (Supplementary File S2), suggesting that *MaXTH*s are likely hydrophilic. The predicted putative localization of the *M. alba XTH* was prevalent in the extracellular region, while a small number were located in the plasma membrane, mitochondrion, cytoplasmic, vacuole, and nuclear region (Table 1).

### 3.2. Gene Structure, Conserved Domain, and Motif Analysis

**Figure 1:**
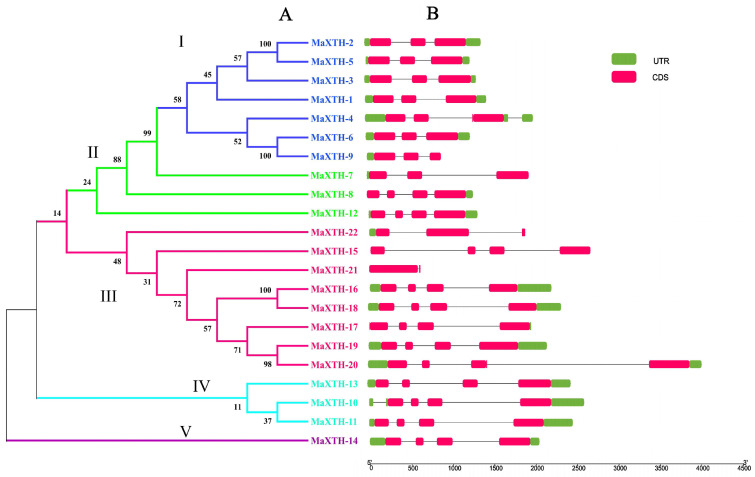
Phylogenetic relationship and gene structure of *MaXTH* gene family. (**A**) Phylogenetic tree of the 22 *Morus alba XTH* gene family (**B**) gene structure of the *MaXTH* genes. Pink color: CDS (coding sequence region); Green color: UTR (untranslated region); I–V: *MaXTH* gene family classification.

The structural diversity of the 22 *MaXTH* genes was investigated by determining their exon and intron structures through the alignment of their genomic and CDS sequences using the TBtool software. In addition, a phylogenetic tree was constructed using the complete *MaXTH* protein sequences, depicted with the distribution of introns and exons (Figure 1). The resulting phylogenetic analysis revealed the categorization of *MaXTH* genes into five distinct groups: I, II, III, IV, and V, constituting 7, 3, 8, 3, and 1 *MaXTH* genes, respectively (Figure 1A). In general, genes that are grouped together share similar structures. For instance, all members of group 1 (*MaXTH-1*, *MaXTH-2*, *MaXTH-3*, *MaXTH-4*, *MaXTH-5*, *MaXTH-6*, and *MaXTH-9*) contained three exons in their coding region, and two introns (Figure 1B). Members of group II also possessed four exons and three introns, except for *MaXTH-7*, which had three exons. *MaXTH* group III members constituted four exons, excluding *MaXTH-21* and *MaXTH-22*. Moreover, *MaXTH-21* contained no intron. Interestingly, *MaXTH-15* had no 5′ or 3′ UTRs.

**Figure 2:**
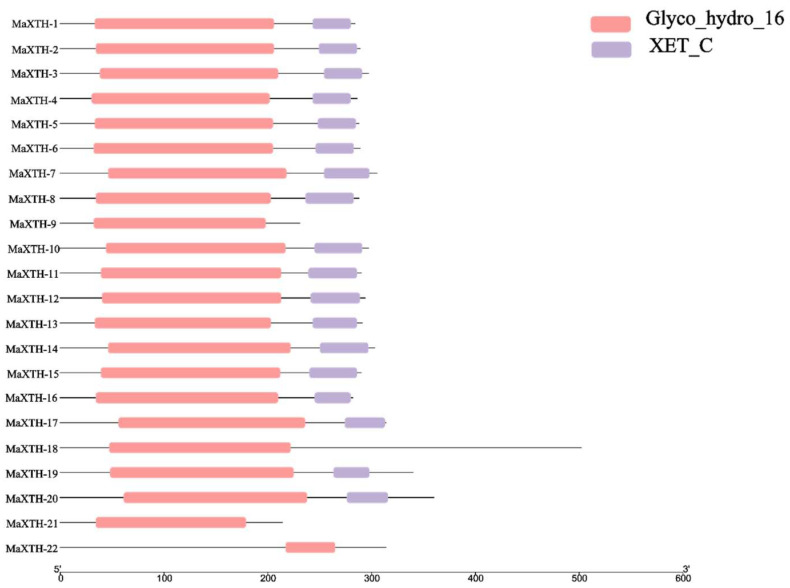
Domain analysis of the *MaXTH* gene family.

**Figure 3:**
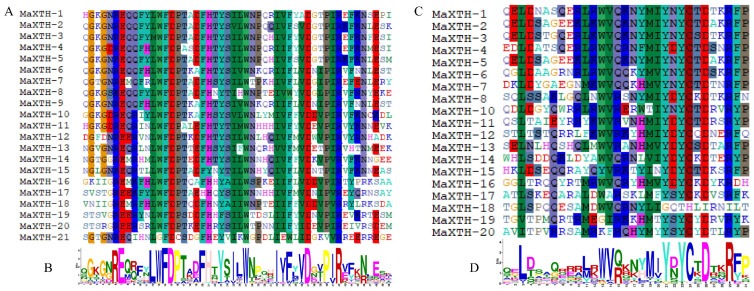
Multiple sequence alignment of domains from MaXTH proteins. (**A**) Multiple alignments of Glyco_hydro_16 domains, (**B**)sequence logo of the Glyco_hydro_16 domains. (**C**) Multiple alignments of XET_C domains (**D**) sequence logo of the XET_C domains.

The Pfam and CDD databases were employed to investigate the conserved domains of the 22 *MaXTH* genes. From the results obtained, only four genes, *MaXTH-9*, *MaXTH-18*, *MaXTH-21*, and *MaXTH-22*, contained a single conserved domain (Glyco_hydro_16). In contrast, the remaining 16 *MaXTH* genes exhibited the presence of both Glyco_hydro_16 and XET_C conserved domains, as illustrated in Figure 2. MaXTH proteins were further characterized by predicting their potentially conserved motifs employing the MEME online software. The differences between the *MaXTH* genes were analyzed through multiple sequence alignment. The findings revealed that 21 members of MaXTH proteins exhibited a typical highly conserved Glyco_hydro_16 domain (Figure 3A). This domain comprised 50 amino acid letters, as illustrated by the sequence logo (QGKGNREQRFYLWFDPTADFHTYSILWNPQHIVFYVDGVPIRVFKNLESK). Though all the 22 XTH proteins identified in this study contained the Glyco_hydro conserved domain by searching the Pfam database, MaXTH 22 lacked the conserved motif sequences. In addition, 19 MaXTHs were identified to consist of the XEC_T domain represented by 31 amino residues (QELDSAQERRLKWVQKNYMIYBYCTDTKRFP).

### 3.3. Evolutionary Relationship of the MaXTH Proteins

**Figure 4:**
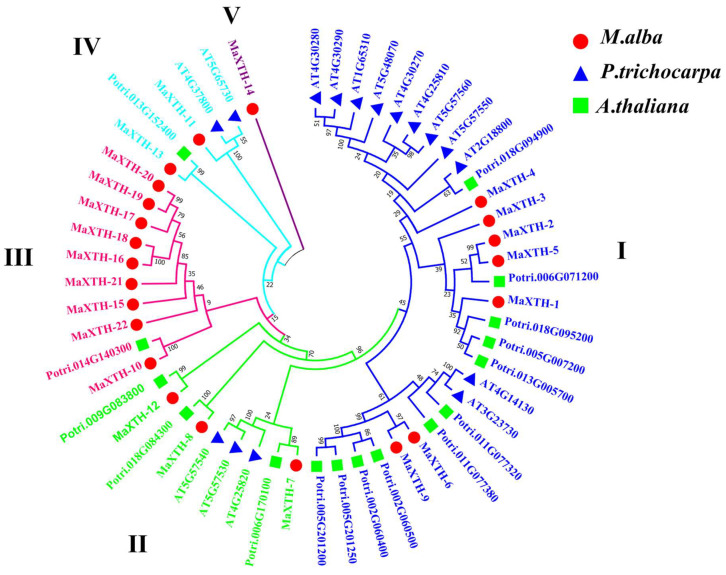
Maximum likelihood Phylogenetic relations of *XTH* protein family in *Morus alba* (red circles), *Populus trichocarpa* (blue triangle), and *Arabidopsis thaliana* (green square). I–V; major clusters.

To comprehend the evolution and classification of MaXTH proteins, a phylogenetic tree based on the maximum likelihood method was constructed using the 22 MaXTH protein sequences with other XTH sequences from *Arabidopsis thaliana* (16) and *Populus trichocarpa* (15). From the results obtained, MaXTH proteins formed 5 different groups with XTH proteins from *A*. *thaliana* and *P*. *trichocarpa* (Figure 4). Groups I, II, III, IV, and V contained 7, 3, 9, 2, and 1 member, respectively. Generally, the phylogenetic analysis depicts that *MaXTH* genes clustered with XTH orthologs from *P*. *trichocarpa* and *A*. *thaliana*. Among the five groups, group 1 was the largest, which comprised 11 members each from *A*. *thaliana* and *P*. *trichocarpa*. Group III was the second largest but mainly consisted of *MaXTH* genes and only one XTH ortholog from *P*. *trichocarpa*. In addition, Group V contained only *MaXTH 14* and did not cluster with any of the XTHs from *A*. *thaliana* and *P*. *trichocarpa*. The non-correlation of the Group 5 member and the limited association between *MaXTH* genes and *P*. *trichocarpa* XTH proteins within Group III suggests that the MaXTH proteins present in the branch might be less evolutionarily conserved or more primitive.

### 3.4. Cis-Acting Regulatory Elements of XTH Genes from Morus alba

**Figure 5:**
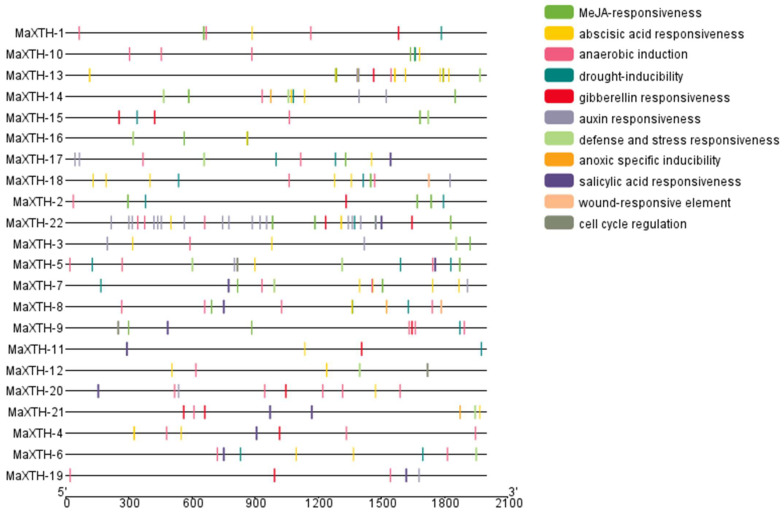
Cis-elements predicted within the 2 kb sequences upstream of the *M. alba XTH* gene promoters. Cis-acting elements, with distinct colors in each box, denote different promoters.

To comprehend the transcriptional regulation of *MaXTH* genes, the 2000 bp promoter sequence of all the *MaXTH* genes was retrieved from the *M*. *alba* genome and analyzed using the PlantCare online database. The data obtained infer that *MaXTH* promoters possessed several cis-regulatory elements involved in processes such as stress response, hormone regulation, and cell development (Figure 5). Defense and stress-responsive cis-elements were identified for 10 *MaXTH* genes, including *MaXTH-3*, *MaXTH-5*, *MaXTH-6*, *MaXTH-7*, *MaXTH-12*, *MaXTH-13*, *MaXTH-14*, *MaXTH-15*, *MaXTH-16* and *MaXTH-21*. In terms of hormone-responsive elements, 11, 14, 10, and 13 MaXTH genes contained salicylic acid-responsive elements, auxin-responsive elements, Methyl jasmonate (MeJa)-responsive regulatory elements, and gibberellin-responsive elements.

Drought-inducible response elements were detected in 13 *MaXTH* genes. The promoter sequences of two *MaXTH* genes constituting *MaXTH-8* and *MaXTH-18* exhibited cis-regulatory elements associated with wound responsiveness. The promoter sequences of two *MaXTH* genes constituting *MaXTH-8* and *MaXTH-18* exhibited cis-regulatory elements associated with wound responsiveness. Anaerobic induction-responsive elements were abundantly detected across all 22 *MaXTH* genes. Other *MaXTH* genes, including *MaXTH-5*, *MaXTH-9*, *and MaXTH-12*, were identified to contain cell cycle response elements that are associated with cell development. These results inferred that *MaXTH* genes participate in diverse biological processes and exhibit responsiveness to different biotic and abiotic stress factors.

**Figure 6:**
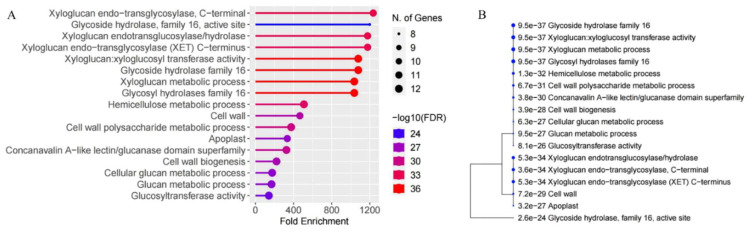
Gene ontology (GO) annotation and functional clustering. (**A**) GO analysis of the 22 *XTH* genes from *Morus alba* and (**B**) A hierarchical clustering tree summarizing the correlation among significant pathways listed in the enrichment tab. Pathways with many shared genes are clustered together. Bigger dots indicate more significant *p*-values.

Gene ontology (GO) analysis was conducted to elucidate the roles of *MaXTH* genes. The proteins encoded by *MaXTH* gene members exhibited both xyloglucan xyloglucosyl transferase and hydrolase activities (Figure 6A and Supplementary File S3). In addition, *MaXTH* genes were observed to play crucial roles in various biological activities, including the organization of the cell wall, the biogenesis of the cell wall, and processes related to xyloglucan metabolism. 

Further, GO analysis revealed that certain members of *MaXTH* genes were localized in the apoplast and cell wall regions. This observed localization aligns with the predictions made through subcellular localization analysis (Table 1). Notably, *MaXTH* genes are implicated in cellular glucan metabolic processes and carbohydrate metabolic processes. Additionally, the hierarchical clustering of the functional enrichment pathways depicted in Figure 6B reveals that *MaXTH* genes collaborate to perform various functions.

### 3.5. Chromosomal Localization, Circos, and Synteny Analyses

**Figure 7:**
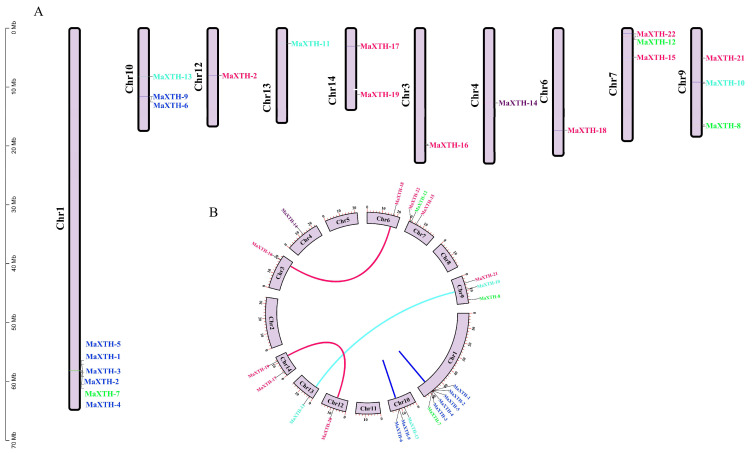
(**A**) Chromosomal localization pattern of *MaXTH* genes. The scale bar on the left represents the length of the chromosomes. (**B**) The collinearity analysis of *MaXTH* genes is represented via a circos map, exhibiting the relationships among the gene pairs. Gene pairs are represented by pink, cyan, and blue lines, while distinct colored labels outside the chromosomes denote gene names belonging to the same phylogenetic family.

The chromosomal positions of *XTH* genes in *M. alba* were located through genome annotation in the TB tools. As depicted in Figure 7, the 22 *MaXTH* genes were unevenly distributed among the chromosomes across the genome of *M. alba* (Figure 7). The most significant *MaXTH* genes were positioned on chromosome one with six members. Three *MaXTH* genes were allotted on chromosomes 7, 9, and 10, and two on chromosome 14. Chromosomes 3, 4, 6, 12, and 13 contained one *MaXTH* each. Interestingly, no *MaXTH* genes were found in chromosomes 2, 5, 8, and 11 (Figure 7A). Based on the phylogenetic relationships of the MaXTH protein sequences (Figure 1A), a total of five *MaXTH* gene pairs were identified, as shown in the circos map (Figure 7B). It was discovered that three (*MaXTH-10* and *MaXTH-11*, *MaXTH-16* and *MaXTH-18*, *MaXTH-19*, and *MaXTH-20*) and two pairs (*MaXTH-9* and *MaXTH-6*, *MaXTH-2* and *MaXTH-5*) of the *MaXTH*s gene pairs belonged to inter-chromosomal and intra chromosomal segments (Figure 7B). Furthermore, the gene pairs were associated with the same phylogenetic group. *MaXTH-16* and *MaXTH-18*, *MaXTH-19* and *MaXTH-20* belonged to group III, *MaXTH-10* and *MaXTH-11* were in group IV (Figure 1), whereas *MaXTH-9* and *MaXTH-6*, *MaXTH-2* and *MaXTH-5* were affiliated to group I.

**Figure 8:**
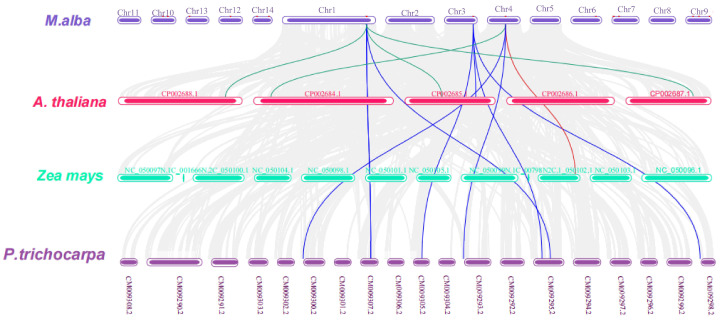
Collinearity analysis of *XTH* genes from *M. alba* and other plant species: *Populus trichocarpa*, *Arabidopsis thaliana,* and *Zea mays*. The presented data delineate *XTH* syntenic gene pairs through distinct colored lines: blue lines signify pairs between *Morus alba* and *Populus trichocarpa*, green lines denote pairs between *M. alba* and *Arabidopsis thaliana*, and a red line signifies pairs between *M. alba* and *Zea mays*. Additionally, grey lines elucidate the presence of orthologous genes of *Morus alba* shared with three other plant species.

To delve deeper into the evolutionary correlations and genetic linkage occurrences within the *XTH* gene family, a systemic map of *XTH* genes across *M*. *alba* and three additional species, encompassing one monocotyledonous (*Zea mays)* and two dicotyledonous (*Populus trichocarpa* and *Arabidopsis thaliana*) plants were constructed using TBtools software (Figure 8).

According to the results of the collinearity analysis, 12 covariate pairs were generally discovered. Among them, 7 pairs were identified in *Populus trichocarpa* (represented in blue lines), 4 pairs in *A. thaliana* (represented in green lines), and only one pair was observed in *Zea mays* (represented in red lines). Furthermore, *MaXTH-14* displayed a significant degree of collinearity with three comparable species (two in *P*. *trichocarpa* and one each in *A*. *thaliana* and *Zea mays*), inferring that *MaXTH* genes displayed greater evolutionary differences in *P*. *trichocarpa* in contrast to *A*. *thaliana*, and *Zea mays*.

### 3.6. Expression Profiling of MaXTH Genes under Different Magnesium Treatments and qRT-PCR Validation

**Figure 9:**
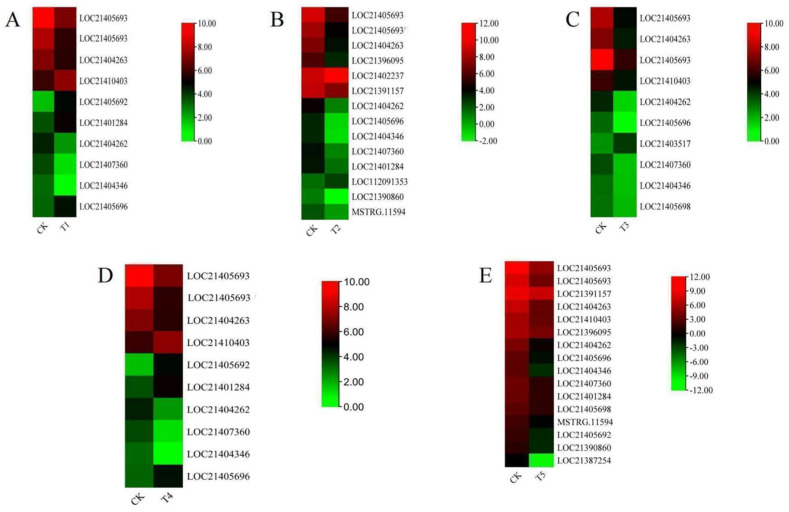
Heatmap of the relative gene expression pattern of the *XTH* gene family based on gene relative expression in *Morus alba* under different magnesium treatments. (**A**) T1; 0 mM, (**B**) T2; 1 mM, (**C**) T3; 2 mM, (**D**) T4; 6 mM and (**E**) T5; 9 mM. CK represents the optimum concentration of magnesium for *M*. *alba* growth (3 mM). From red to green, show the concentration level of the gene expression.

Several studies have reported that the *XTH* gene family plays an important role in plant response to abiotic stresses [17,18,20]. To validate these accessions, the expression patterns of *M*. *alba XTH* genes responding to different treatments of magnesium concentrations were investigated by RNA-seq. RNA from *M*. *alba* leaf tissues was sampled on day 20. The *XTH* genes with expression changes according to the significant differential expression standard (|log2 (Fold change)| ≥ 1 and false discovery rate (FDR) < 0.05) were analyzed. The results from our investigation exhibited different expression levels of *XTH* genes at various concentrations (Figure 9A–E). At the least concentration of 0 mM (T1), 10 *MaXTH*s were identified, among which six were significantly downregulated. Four *XTHs* genes, including LOC21410403, LOC21405693, and LOC21401284, showed higher expression concentrations (Figure 9A).

In the 1 mM (T2), we detected 14 *MaXTH*s, of which LOC21462237 was upregulated and had the highest expression level (Figure 9B). In contrast, the remaining 3 *XTH* genes showed downregulated expressions (LOC21390860, LOC21404346, and LOC21405696). *M*. *alba* treatment with 2 mM (T3) revealed 10 *MaXTH*s (Figure 9C). For 6 mM treatments (T4), it was observed that 3 *XTH*s (LOC21404346, LOC21404262 and LOC21407360) were low in expression (Figure 9D). In contrast, four *XTH*s (LOC21410403, LOC21405693, LOC210401284 and LOC21405696) were upregulated (Figure 9D). At the excess Mg of 9 mM (T5), all 10 *MaXTH* genes identified were significantly upregulated (Figure 9E), with LOC21387254 and LOC21404346 being down-regulated.

**Figure 10:**
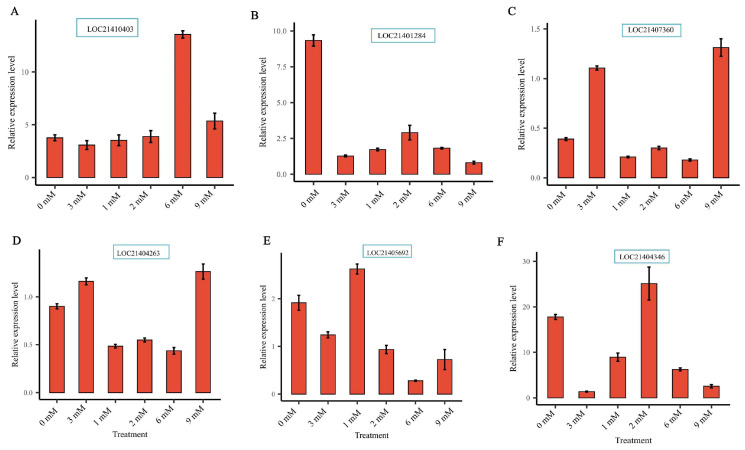
The verification of relative expression levels of six *MaXTH* genes by RT-qPCR under different magnesium treatments. (**A**) LOC21410403 gene, (**B**) LOC21401284 gene, (**C**) LOC21407360 gene, (**D**) LOC21404263 gene, (**E**) LOC21405692 gene, and (**F**) LOC21404346 gene. Bars are means of three replicates.

Furthermore, six *MaXTH* genes, including *MaXTH-17* (LOC21410403), *MaXTH-13* (LOC21401284), *MaXTH-21* (LOC21407360), *MaXTH-1* (LOC21405692), *MaXTH-6* (LOC21404263) and *MaXTH-10* (LOC21404346) in response to Mg stresses, were selected to verify their relative expression level in mulberry leaves through qRT-PCR analysis. The results reveal that the selected genes could be expressed in the mulberry leaves, proving the reliability of the *XTH* genes identified by the transcriptome data (Figure 10A–E). LOC21410403 (*MaXTH-17*) exhibited a low expression level at 3 mM (CK; optimum concentration for *M*. *alba* growth); however, it was highly expressed at an elevated concentration of 6 mM (Figure 10A). For LOC21401284 (*MaXTH 13*), the highest expression level was observed at Mg deficiency (0 mM) (Figure 10B). LOC21407360 (*MaXTH-21*) was moderately expressed at 0 mM but was highly expressed at 3 and 9 mM (Figure 10C). The expression of LOC21404263 (*MaXTH-6*) was highly expressed in 0. 3 and 9 mM Mg treatments (Figure 10D). For the LOC21405692 (*MaXTH-10*), the highest expression level was observed at 3 mM, and the expression level was reduced at 6 mM concentration (Figure 10E). Finally, the expression of LOC21404346 (*MaXTH-1*) was higher in 0 and 2 mM concentrations (Figure 10E). Altogether, our findings confirm that the *XTH* genes family is highly present in the *M*. *alba* genome and expressed in *M. alba* leaf tissues in response to Mg imbalances. This outcome strongly suggests that the *XTH* gene family is important in *M*. *alba* development and nutritional regulation.

## 4. Discussion

Mulberry (*M. alba*) is a plant of considerable economic importance, yet its growth and development are influenced by various abiotic factors such as Mg deficits [5]. Mg serves diverse functions in biological systems [48]. Consequently, gaining insights into how plants respond to both Mg deficiency and excess at the genomic level is essential for effective plant nutrient management. Past research indicates that plants have evolved sophisticated regulatory mechanisms, engaging specific gene families like xyloglucan endotransglucosylase/hydrolases (*XTH*s) [10] to facilitate their adaptation to Mg stress.

*XTH*s represent a category of plant enzymes responsible for regulating xyloglucan crosslinking within cell walls, playing a pivotal role in the control of plant growth and development [49,50]. The role of XTH genes is not only limited to cell wall elongation but also plays a part in plant responses to various environmental stresses. The *XTH* gene family has been identified across diverse plant species, such as *A. thaliana* [51], wheat [52], grapevine [53], rice [21], peanut [17], barley [54], sweet potato [20] and poplar [55]. Within the scope of this investigation, we present the discovery and characterization of the *XTH* gene family within the *M*. *alba* genome. This includes exploring their phylogenetic relationships, conserved motifs, gene structures, cis-acting regulatory elements, and gene expression patterns in response to Mg starvation, low or high concentrations.

Based on the *M. alba* genome, 22 *XTH* genes were identified based on our strictest identification workflow and labeled as *MaXTH-1* to *MaXTH-22*. The number of identified *XTH* genes was notably less compared to various other species, including tobacco (56), wheat (71), *Solanum lycopersicum* (37), and *Glycine max* (61) [11,18,22,52]. It is widely acknowledged that the functional attributes of genes are intricately linked to their structural and physicochemical characteristics [20,56]. In this study, the 22 *MaXTH* protein members displayed significant disparities with respect to protein sequence length, molecular weight, isoelectric point (pI), and intron and exon distributions (Table 1). This variation implicates a high diversity among *XTH* family members in *M*. *alba*. Additionally, most *MaXTH* genes were predicted to be in the extracellular space, while a few were in the plasma membrane, vacuole, mitochondrion, and nuclear region. This is contrary to previous reports for other *XTH* protein members in other plant species, where the majority of the *XTH* proteins were in the plasma membrane rather than the extracellular space and other locations [22,53,55]. Further, phylogenetic analysis indicated that *MaXTH* protein families were clustered into five groups (Figure 1), similar to those observed for *XTH* proteins from sweet cherry [26]. Interestingly, the *MaXTH* proteins belonging to the same group demonstrated similar gene structures (Figure 1) and conserved sequence expression, which is consistent with previously documented literature [11,20], suggesting that *XTH* members within the same group may exhibit analogous functionalities. Moreover, most of the *MaXTH* genes demonstrate the presence of two main conserved domains (Glyco_hydro_16 and XET_C domain) (Figure 2). Nevertheless, *MaXTH-9*, *MaXTH-18*, *MaXTH-21*, and *MaXTH-22* lacked the XET_C domain. This absence suggests a potential evolutionary divergence, indicating a loss of the XET_C domain during the evolutionary trajectory of *XTH* proteins in *M*. *alba*.

Phylogenetic distribution of *XTH* proteins from *M*. *alba*, *A*. *thaliana*, and *P*. *trichocarpa* revealed that *MaXTH* genes could be categorized into five groups (group I–V) (Figure 4). Earlier studies have documented the categorization of *XTH* gene families into distinct groups in various plant species. In tobacco, for instance, eight family groups were identified [22], while three groups were observed in peanut [17], barley [54], and sweet potato [20]. Poplar, on the other hand, exhibited four distinct groups [55]. The *MaXTH* genes were observed to cluster better with *XTH* proteins from *P*. *trichocarpa* than *A*. *thaliana*, implying a closer evolutionary relationship between *XTH* proteins in *M*. *alba* and those of *P*. *trichocarpa* rather than *A*. *thaliana*. According to chromosomal localization analysis, it was observed that *MaXTH*s were heterogeneously distributed on 10 out of the 14 chromosomes of *M*. *alba* (Figure 7). Further investigation revealed five gene pairs among the *XTH* gene families in the *M*. *alba* genome. Previous research has indicated that a set of gene functions exhibit high conservation across various plant species [57,58]. Consequently, it is imperative to identify true orthologs in different plant species through the application of synteny analysis. The results obtained from the synteny analysis depicted a significant degree of synteny between the *M*. *alba* genome and those of *P*. *trichocarpa* and *A*. *thaliana*, exhibiting 7 and 4 synthetic blocks of *MaXTH* between *P*. *trichocarpa* and *A*. *thaliana*, respectively. In contrast, one synthetic block was identified between *Zea mays* (Figure 8).

Cis-regulatory elements are essential for regulating gene expression. The comprehension of cis-regulatory elements within the promoter region of genes has the potential to clarify the roles and regulatory mechanisms of specific genes that engage in collaborative interactions with other genes [59,60]. Investigating the cis-regulatory elements of the 22 *MaXTH* exhibited a number of core promoters involved in hormone responsiveness (Abscisic acid, Salicylic acid, MeJA, Gibberellin), stress responsiveness (stress defense, drought, anoxic, and anaerobic inducibility), growth and development elements (Figure 5). *MaXTH* promoters contain a variety of elements that respond to environmental and plant hormone stimuli, which might indicate various regulatory or functional mechanisms in response to biotic and abiotic stress factors [20,61]. Besides, there were significant variations in terms of type and quantity, and certain elements related to metabolism and gene expression were unique to specific *MaXTH* genes. The structural variations of *MaXTH* proteins could result in modifying protein functions. Several studies have demonstrated that plant *XTH* proteins have essential roles in plant growth, development, and stress resistance. The presence of numerous cis-elements identified in the promoter region of the *M*. *alba XTH* genes suggests that the *XTH* genes within *M*. *alba* possess the capability to adapt to diverse modifications in the plant, particularly responsiveness to several hormones and numerous stress response elements (anaerobic and anoxic specific inducibility).

Analyzing gene expression profiles can advance our understanding of *XTH*s functions in *M*. *alba* growth and development. Analysis of transcriptome data at day 20 after the various magnesium treatments indicated that several *XTH*s were expressed in response to the treatments (Figure 9A–E). *XTH* genes, including *MaXTH-17* (LOC21410403), *MaXTH-13* (LOC21401284), *MaXTH-21* (LOC21407360), *MaXTH-6* (LOC21404263), and *MaXTH-10* (LOC21404346) highly expressed at 0, 1, 2, 6, and 9 mM of Mg concentration, respectively (Figure 10A–F) compared to control (3 mM, optimum Mg for *M*. *alba* growth). Meanwhile, *MaXTH-6* (LOC21404263) was downregulated at 2 and 6 mM, while *MaXTH-1* (LOC21405692) exhibited low expression at 6 mM. Prior findings indicate that abiotic stressors can induce transcript-level changes in *XTH* genes. For example, in response to cadmium (Cd) stress, the expression of *BnXTH1*, *BnXTH3*, *BnXTH6*, and *BnXTH15* was observed to be upregulated in *Boehmeria nivea*. Conversely, *BnXTH18*, *BnXTH16*, *BnXTH17*, and *BnXTH5* exhibited notable downregulation under the same Cd stress conditions [62].

Similar contrasting expression patterns of the *XTH* gene family were identified in *Camellia sinensis* under fluorine stress where *CsXTH7*, *CsXTH1*, *CsXTH6*, and *CsXTH1* were upregulated, while that of *CsXTH3* was down-regulated [63]. Additionally, the expression of *PeXTH* experienced a notable upregulation in the roots and leaves of *P*. *euphratica* when exposed to Cd stress [37]. Likewise, under Al stress, *AtXTH15* and *AtXTH14* demonstrated a significant decrease, leading to a reduction in xyloglucan endo transferase (*XET*) activity and consequently enhancing the aluminum tolerance of *A*. *thaliana* [64]. In this study, we observed different expression patterns of *MaXTH* genes. Changes in the expression of *MaXTH* genes can affect cell wall flexibility and strength, which are important factors in stress adaptation. The increased expression of certain *MaXTH* genes might contribute to cell wall remodeling, allowing for better flexibility and adaptation to magnesium stress. Conversely, decreased expression could be associated with a more rigid cell wall structure. These findings indicate the capacity of *MaXTH* genes to provide a defense to the *M*. *alba* plant during magnesium starvation, undersupply, and excess application. Future works in *M. alba* should investigate the functional genomic validation of these identified *XTH* genes and how they regulate Mg nutrition.

Taken together, the results of this research offer novel insights into the expression of *MaXTH* genes under different Mg concentrations. It could be inferred that the *MaXTH*s might exhibit heightened functionalities related to the cell wall in stressful conditions through interaction with xyloglucan. However, additional molecular and genetic research is required to confirm their roles.

## 5. Conclusions

In this current investigation, an extensive examination of the *M*. alba *XTH* gene family was conducted. The results from the investigation successfully identified and further characterized a total of 22 *MaXTH* genes. These genes were subsequently categorized into five groups (I to V) based on their phylogenetic relationships. Gene structure and motif composition were observed to be consistent within each group. A thorough analysis of gene synteny uncovered evidence of evolutionary relationships among *XTH* genes in *M*. *alba* and three other species, *A*. *thaliana*, *P*. *trichocarpa*, and *Zea mays.* Gene ontology analysis revealed that *MaXTH*s are responsible for encoding proteins demonstrating both xyloglucan xyloglucosyl transferase and hydrolase activities. Moreover, *MaXTH*s actively contribute to the processes of cell wall biogenesis, as well as the metabolic pathways associated with xyloglucan and carbohydrates. Furthermore, specific Cis-acting regulatory elements detected in the promoter region of *MaXTH* genes suggest their potential involvement in various biological processes such as development, phytohormone responses, and stress adaptation. Moreover, investigating the expression profiles of *MaXTH* genes within leaf tissues exposed to different magnesium concentrations revealed diverse patterns of gene expression. Collectively, the findings from this research provide valuable insights into the functions of *XTH* genes within *M*. *alba* and present a better understanding of how mulberry plants respond to various magnesium treatments.

## Figures and Tables

**Table 1:** Physiological characteristics of *XTH* gene family in Morus alba.

Gene ID	Gene Name	Chromosome	CDS (bp)	Protein Length (aa)	Exons	pI	Protein Molecular Weight (kDa)	Sublocalization
LOC21405692	*MaXTH-1*	1	855	284	3	8.15	32.220	Extracellular
LOC21405693	*MaXTH-2*	1	870	289	3	6.31	32.211	Extracellular
LOC21405698	*MaXTH-3*	1	894	297	3	5.95	33.323	Extracellular
LOC21405697	*MaXTH-4*	1	861	286	3	4.96	32.655	Extracellular
LOC21405696	*MaXTH-5*	1	867	288	3	6.21	32.501	Extracellular
LOC21404263	*MaXTH-6*	10	870	289	3	8.94	32.771	Extracellular
LOC21405699	*MaXTH-7*	1	918	305	3	6.6	35.306	Extracellular
LOC21387185	*MaXTH-8*	9	867	288	4	8.96	32.893	Extracellular
LOC21404262	*MaXTH-9*	10	813	231	4	5.28	25.884	Extracellular
LOC21404346	*MaXTH-10*	13	894	297	5	8.87	34.529	Extracellular
LOC21391157	*MaXTH-11*	9	873	290	4	6.24	33.270	Extracellular
LOC21387254	*MaXTH-12*	7	885	294	4	8.56	34.306	Extracellular
LOC21401284	*MaXTH-13*	10	885	291	4	5.71	33.172	Extracellular
LOC21396095	*MaXTH-14*	4	912	303	4	4.72	35.292	Extracellular
LOC21390452	*MaXTH-15*	7	873	290	4	5.09	33.165	Extracellular
LOC21405370	*MaXTH-16*	3	849	282	4	9.34	32.603	Extracellular, Mitochondrial
LOC21410403	*MaXTH-17*	14	945	314	4	7.67	35.265	Extracellular, Vacuole
LOC21403517	*MaXTH-18*	6	1509	502	4	9.74	56.843	Plasma membrane
LOC21402237	*MaXTH-19*	14	1023	340	4	6.27	38.748	Extracellular
LOC21391267	*MaXTH-20*	12	1083	360	4	8.73	41.304	Extracellular
LOC21407360	*MaXTH-21*	9	645	214	1	5.52	24.071	Cytoplasmic, Extracellular
LOC21390860	*MaXTH-22*	7	945	314	3	5.68	35.599	Cytoplasmic, Nuclear

## Data Availability

Data are contained within the article and Supplementary Materials. Other request can be directed to the corresponding author(s).

## Supplementary Materials

The following supporting information can be downloaded at: https://www.mdpi.com/article/10.3390/plants13060902/s1, Supplementary File S1: Gene primer; Supplementary File S2: Physiology data XTH; Supplementary File S3: enrichment_all.
