# Supplementary material for: Genome-Wide Analysis of the Xyloglucan Endotransglucosylase/Hydrolase (XTH) Gene Family: Expression Pattern during Magnesium Stress Treatment in the Mulberry Plant (Morus alba L.) Leaves
Source: Plants (Basel). 2024 Mar 21;13(6):902. doi: 10.3390/plants13060902 (PMC10975095; doi:10.3390/plants13060902)
Supplement: Supplementary file 1 [file plants-13-00902-s001.zip › Supplemental file S1Gene primer.pdf]

Table 2. RT-qPCR primers for the validation of *Morus alba* XTH gene family

Gene name	Primer sequence (5'-3')
LOC21401284 (MaXTH-13)	F ACTGGAGAGCCCTATTCGGT<br>R GCCCATGGCTTGGTCTTTTG
LOC21404346 (MaXTH-10)	F TGTGGACGATGTGCCAATCA<br>R TTGCCTTGGGTTGCACAGTA
LOC21404263 (MaXTH-6)	F AAGCTCCTTTCACTGCCTCC<br>R TGAGGGAAGCGTTTGGAGTC
LOC21407360 (MaXTH-21)	F CCAGGACGAGATCGACTTCG<br>R CCTTCTCTCCGCTCCTCTCT
LOC21405692 (MaXTH-1)	F TTGGGGAATTTGAGTGGCGA<br>R ACGCCGATTGGTTCTGAGTT
LOC21410403 (MaXTH-17)	F AGCACCACCAGTACAGCATC<br>R AAACGACACCACAAAAGGCG
